# Modifiable and non-modifiable epidemiological risk factors for acne, acne severity and acne scarring among Malaysian Chinese: a cross-sectional study

**DOI:** 10.1186/s12889-021-10681-4

**Published:** 2021-03-27

**Authors:** Yee-How Say, Anna Hwee Sing Heng, Kavita Reginald, Yi Ru Wong, Keng Foo Teh, Smyrna Moti Rawanan Shah, Yang Yie Sio, Yu Ting Ng, Sri Anusha Matta, Sze Lei Pang, Fook Tim Chew

**Affiliations:** 1 Department of Biological Sciences, Faculty of Science, National University of Singapore, Allergy and Molecular Immunology Laboratory, Lee Hiok Kwee Functional Genomics Laboratories, Block S2, Level 5, 14 Science Drive 4, Lower Kent Ridge Road, Singapore, 117543 Singapore; 2 Department of Biological Sciences, School of Medical and Life Sciences, Sunway University, 47500 Petaling Jaya, Selangor Malaysia

**Keywords:** Acne, Acne severity, Acne scarring, Dietary habits, Epidemiology, Risk factors

## Abstract

**Background:**

Acne vulgaris, a highly prevalent multifactorial inflammatory skin disease, can be categorised into different severity and scarring grades based on the type, number, and severity of lesions. While many epidemiology studies have investigated the risk factors for acne presentation, fewer studies have specifically studied the risk factors for acne severity and scarring. Therefore, this study investigated the prevalence of acne, acne severity and scarring grades, and their associated non-modifiable and modifiable epidemiological risk factors among Malaysian Chinese.

**Methods:**

A total of 1840 subjects (1117 cases/723 controls) completed an investigator-administered questionnaire as part of a cross-sectional study, which include socio-demographics, familial history, lifestyle factors, dietary habits, and acne history. Acne cases were further evaluated for their severity (*n* = 1051) and scarring (*n* = 1052) grades by a trained personnel.

**Results:**

Majority of the acne cases (up to 69%) had mild acne or Grade 1/2 scarring, while 21.6% had moderate/severe acne and 5.5% had Grade 3/4 scarring. Males had significantly higher risk of presenting with higher grades of acne scarring. Those who had acne, regardless of severity and scarring grades, had strong positive familial history (either in parents and/or sibling). Frequent consumption (most or all days) of foods that are commonly consumed during breakfast (butter, probiotic drinks, cereals and milk) decreased the risk for acne presentation and higher acne scarring, while periodic consumption (once/twice per week) of nuts and burgers/fast food decreased the risk for higher acne severity. Alcohol drinking was significantly associated with increased risk for acne presentation, while paternal, parental and household smoking were associated with reduced risk of more severe acne.

**Conclusions:**

In conclusion, positive familial history is a strong predisposing factor in influencing acne presentation, severity and scarring. Frequent consumption of foods that are commonly consumed during breakfast is protective against acne presentation.

**Supplementary Information:**

The online version contains supplementary material available at 10.1186/s12889-021-10681-4.

## Background

Acne vulgaris (henceforth acne) is a chronic inflammatory skin disorder with a multifactorial pathogenesis. It is the eighth most common skin disease worldwide, with the 2010 Global Burden of Disease Study estimating a global prevalence of acne (for all ages) of 9.38% [1]. However, in different countries and among different age groups, the prevalence of acne varies, with estimates ranging from 35% to close to 100% of adolescents having acne at some point [2]. Apart from the discomfort due to the clinical symptoms of acne, patients may experience other negative psychosocial impacts, like higher unemployment rates [3], increased depression and anxiety rates, lower self-esteem, and negative body image [4].

The pilosebaceous unit is well known as the site of acne development. The structure comprises the hair follicle and its connected sebaceous gland, which produces and secretes sebum onto the skin surface via the pore of the hair follicle [5]. Acne lesions develop when the pore of the pilosebaceous unit is blocked or inflamed [6]. The severity of acne is characterized by the number of non-inflammatory closed and open comedones, and inflammatory pustules, papules, and nodules. Cysts, scars, erythema and hyperpigmentation could also be present in more severe acne [6]. For this reason, acne may be present in a wide variety of clinical forms depending on the type, number, and severity of the predominant lesions, and could be categorised into different severity [7] and scarring [8] grades.

The most notable pathophysiological factors that influence the development of acne are increased *Cutibacterium acnes* colonisation of the follicle, sebaceous gland hyperplasia with seborrhoea, abnormal desquamation of sebaceous follicle epithelium (comedogenesis), and, moreover, inflammatory and immunological reactions [5]. Recently, we reviewed that epidemiological risk factors influencing acne presentation and severity include non-modifiable factors like positive genetic predisposition or familial history, and hormonal factors attributed to gender and age; and modifiable factors like lifestyle factors (smoking, alcohol, physical activity) and dietary habits of certain food types/groups [9].

While many epidemiology studies have investigated the risk factors for acne presentation, fewer studies have specifically studied the risk factors for acne severity and acne scarring. To date, Malaysian studies studying acne have only investigated the influence of dietary habits [10, 11], or the impact of acne on the quality of life [12, 13]. Therefore, this study investigated the prevalence of acne, acne severity and scarring grades, and their associated non-modifiable and modifiable epidemiological risk factors among Malaysian Chinese.

## Methods

### Participants and data collection

A cross-sectional study aimed to investigate the epidemiology of acne presentation, severity and scarring in a Malaysian Chinese population was conducted at Universiti Tunku Abdul Rahman (UTAR), Malaysia, in 2016 and 2018, and in Sunway University, Malaysia, in 2019. Due to the unequal percentages of the three main races, there may be a lower probability of sampling among Malays or Indians, which may lead to ascertainment bias. In the event of ascertainment bias, the results obtained from the epidemiological investigation may not be representative of certain racial groups in the population, making it difficult to interpret the results obtained. Further, ascertainment bias will lead to a loss of statistical power, affecting the quality of the results obtained. Dividing the sample population into racial subgroups for analysis was not practical, as the sample sizes for Malays and Indians were small, and there was a lack of statistical power for these comparisons. As such, only the majority racial group, the Chinese were included.

A validated investigator-administered questionnaire was adapted to collect information on demographics, personal and lifestyle habits, medical history, family history, dietary habits, socioeconomic status and acne history [14, 15] (Supplementary Information: Additional file 1). Particularly on the dietary habits, all the foods in question 37 of the original questionnaire [14] were included - except that cooked and raw vegetables, cereals and bread, fast foods and burgers were combined into single categories; olive oil, other dairy, sugar, fizzy/soft drinks were removed; Yakult®/Vitagen®/similar yoghurt drinks was included. Ethical approval was granted from the Scientific and Ethical Review Committee (SERC) of UTAR (Ref. code: U/SERC/03/2016) and Sunway University Research Ethics Committee (Ref. code: SUREC 2019/029). All participants signed informed consent forms, and the study was conducted in accordance with the Declaration of Helsinki.

Participants were assessed for acne presentation and were classified as acne cases or controls using a definition designed to determine if participants have ever had acne. Acne cases had to fulfil at least one of four criteria: (1) they had ever visited a doctor for their acne condition; (2) they had scars (keloids) left by acne/boils; (3) they had moderate or severe acne; (4) they had Grade 3 or 4 scarring. Acne controls had to fulfil all of the following criteria: (1) they had never ever visited a doctor for their acne condition; (2) they never had scars (keloids) left by acne/boils; (3) they had no moderate or severe acne; (4) they had no grade 3 or 4 scarring. After excluding non-Malaysian Chinese, the total sample size was 1943, with 1117 acne cases and 723 acne controls. A total of 103 (5.3%) individuals were excluded from both acne and acne severity analyses, as they did not fulfil the criteria to be classified as an acne case or an acne control.

A dedicated post-doctoral medical staff trained by a dermatologist assessed acne severity and scarring grades on a subset of acne cases, and assessment was cross-validated by the dermatologist who turned up on selected days of collection and performed the assessment side-by-side. There was no discrepancy between the assessments by the post-doctoral staff and dermatologist. Four categories were used for acne severity: (1) Nil, with ≤5 comedones; (2) Mild, either (i) with > 5 comedones and if inflammatory lesions are present, < 25 total acne lesions (both inflammatory and non-inflammatory) or (ii) if only non-inflammatory lesions are present, the subject will be categorised as mild as long as there are > 5 lesions regardless of number of total lesions present; (3) Moderate, with ≥25 total acne lesions (both inflammatory and non-inflammatory) and ≥ 1 inflammatory lesion; and (4) Severe, either (i) with ≥30 total acne lesions, including ≥3 inflammatory lesions, affecting a large area of the face and or (ii) with ≥10 inflammatory lesions regardless of area of face affected. This system was based on the Global Evaluation Acne (GEA) scale [7], with a few modifications. Firstly, as no participant fulfilled the criteria for grade 5 acne, we excluded grade 5 of the GEA scale from this study. Secondly, to make the grading more objective and reproducible, specific counts of non-inflammatory and inflammatory acne lesions were used to grade acne severity. Thirdly, due to the use of specific acne lesion counts, we removed the GEA scale criteria on the area of the face affected by acne lesions from our grading system.

Participants’ acne scarring was classified into 5 scarring grades: (1) Grade 0, or no acne scars; (2) Grade 1, with shallow scars not visible from a distance affecting less than one quarter of the face; (3) Grade 2, with slightly deeper scars that are slightly visible from a distance affecting more than one quarter but less than half of the face; (4) Grade 3, with even deeper scars visible from a distance affecting at least half of the face; and (5) Grade 4, with the deepest scars that are clearly visible from a distance affecting almost the entire face. This grading system was adapted from the Qualitative Global Scarring Grading System (QGS [8];) and a few changes were made to make the system more suitable for an epidemiological setting. Firstly, unlike grade 1 of the QGS, we did not consider erythema or hyperpigmentation marks in our acne scar grading system, as these types of marks often diminish and disappear with time [8]. Further, as erythema and hyperpigmentation marks on the face may appear due to causes other than acne [16], such marks were excluded from our grading system. Another change made to the QGS was the consideration of the surface area of the face affected by the scars. This additional criterion was added to reduce the number of patients that fall between two scarring grades.

### Statistical analysis

The data was cleaned by removing any invalid or inconsistent responses, and then analysed using the IBM® *SPSS*® Statistics software (IBM Inc., NY). Binary logistic regression was used to model the association between acne presentation and potential risk factors. For acne severity comparisons, moderate and severe acne were combined into a single category for analysis due to the small sample size for severe acne. Univariate analyses were first conducted, followed by multivariate analyses adjusting for age and gender. For multivariate adjustment, subjects were categorised into three groups for age (≤19, 20–24 and > 24 years old) and two groups for sex (male, female). Results were presented as odds ratios (OR) with 95% confidence intervals (CI). Statistical significance of results was defined as *p* < 0.05.

## Results

### Prevalence of acne, acne severity grades and acne scarring grades

The distribution of demographic characteristics of the sampled population is presented in Table 1. Participants were aged between 17 and 77 years (median = 21 years, ± SD 4.926; M/F = 1:2.2) and the overall prevalence of acne in the sampled population was 60.7% (males: 33.5%, females: 66.5%). Acne prevalence was the highest among those aged 20 to 24, followed by ≤19 and > 24. There was no significant difference in acne case/control distribution between genders (χ^2^ = 0.722; *p* = 0.396) or age groups (χ^2^ = 4.232; *p* = 0.12). Among the 1117 cases, 1051 and 1052 individuals underwent assessment for acne severity and acne scarring, respectively. Among those assessed, 9.2 and 30.1% had no current acne or current scars and were excluded from severity and scarring analyses, respectively (Table 1). Majority of the acne cases (up to 69%) had mild acne severity and grade 1 scarring, while less than 6% had higher grades of acne severity or scarring (severe or grade 3 and 4 scarring).

**Table 1:** Demographic characteristics of acne cases and controls and prevalence of severity and scarring grades among acne cases

Variable	Case (***n*** = 1117)	Control (***n*** = 723)
**Gender**		
Male	374 (33.5)	228 (31.5)
Female	743 (66.5)	494 (68.3)
Not stated	0 (0.0)	1 (0.1)
**Age**		
> 24	56 (5.0)	52 (7.2)
20 to 24	749 (67.1)	463 (64.0)
≤ 19	312 (27.9)	207 (28.6)
Not stated	0 (0.0)	1 (0.1)
**Acne severity grades (*n*** **= 1051)**		
Nil (no current acne)	97 (9.2)	
Mild	727 (69.2)	
Moderate	224 (21.3)	
Severe	3 (0.3)	
**Acne scarring grades (*n*** **= 1052)**		
Grade 0 (no current scars)	317 (30.1)	
Grade 1	431 (41.0)	
Grade 2	246 (23.4)	
Grade 3	57 (5.4)	
Grade 4	1 (0.1)	

Values are frequencies (%)

### Familial history is a strong predisposing risk factor for acne presentation, while frequent butter and probiotic drink consumption reduces risk of acne

The risk factors that are significantly associated with acne presentation are presented in Table 2. A positive familial history of acne was strongly associated with increased odds for acne presentation, even after controlling for age and gender. Higher risk for acne was observed in individuals with a maternal, paternal, sibling, or at least one parent/sibling with history of acne, compared to those without familial history. Furthermore, when maternal and paternal familial history were analysed together as parental familial history, it was observed that having only one parent with acne increased the odds of acne to 3.663 (Table 2), while having both parents with acne increased the odds of acne to 3.509 (Table 2), suggesting that maternal and paternal familial history did not show additive effects.

**Table 2:** Association of selected familial history, co-morbidities and lifestyle habits with acne presentation

Variable	Unadjusted	***p***	Adjusted^**a**^	***p***
	OR (95% CI)		OR (95% CI)	
***A. Socio-demographic factors***				
**Age**				
> 24	1.00	–	1.00	–
20–24	1.502 (1.012–2.227)	0.043*	1.499 (1.009–2.222)	0.045*
≤ 19	1.401 (0.923–2.123)	0.114	1.397 (0.921–2.119)	0.116
**Household number**				
≤ 2	1.00	–	1.00	–
2–4	2.037 (1.172–3.534)	0.012*	1.894 (1.079–3.322)	0.026*
> 4	2.146 (1.247–3.690)	0.006**	1.984 (1.136–3.460)	0.016*
***B. Familial history***				
**Maternal Acne**				
Yes	2.872 (2.253–3.660)	< 0.001***	2.861 (2.239–3.655)	< 0.001***
No	1.00	–	1.00	–
**Paternal Acne**				
Yes	3.314 (2.555–4.298)	< 0.001***	3.319 (2.553–4.315)	< 0.001***
No	1.00	–	1.00	–
**Parental Acne**				
None	1.00	–	1.00	–
Only 1 parent	3.559 (2.494–5.076)	< 0.001***	3.663 (2.564–5.236)	< 0.001***
Both	3.497 (2.618–4.651)	< 0.001***	3.509 (2.625–4.717)	< 0.001***
**Sibling Acne**				
Yes	3.187 (2.544–3.992)	< 0.001***	3.167 (2.527–3.968)	< 0.001***
No	1.00	–	1.00	–
**Familial Acne**				
None	1.00	–	1.00	–
At least 1 parent/sibling	4.184 (3.279–5.348)	< 0.001***	4.202 (3.289–5.348)	< 0.001***
***C. Lifestyle factors***				
**Alcohol drinking**				
Non-drinker	1.00	–	1.00	**–**
Drinker	1.292 (1.071–1.560)	0.008**	1.264 (1.045–1.529)	0.016*
***D. Dietary habits***				
**Butter consumption**				
Never/ occasionally	1.00	–	1.00	**–**
Once or twice/week	0.917 (0.724–1.163)	0.476	0.912 (0.717–1.157)	0.446
Most or all days	0.672 (0.460–0.983)	0.041*	0.666 (0.455–0.975)	0.036*
**Probiotic drink consumption**				
Never/ occasionally	1.00	–	1.00	**–**
Once or twice/week	1.189 (0.931–1.520)	0.165	1.185 (0.926–1.515)	0.178
Most or all days	0.723 (0.523–0.999)	0.049*	0.701 (0.506–0.973)	0.034*

Values are odds ratio (95% CI)

*p < 0.05 ** *p* ≤ 0.009 *** *p* ≤ 0.000

^a^Adjusted for age and gender

Apart from familial history, age and household number were two socio-demographic factors significantly associated with acne presentation. The risk for acne was significantly higher in those aged 20–24 relative to those > 24, and in those who came from a household with numbers ≥2 relative to those < 2 (Table 2). The significant association between household number and acne presentation remained even after further correction for parental acne, (adjusted OR = 2.092, 95% CI: 1.020–4.292, *p =* 0.044 for 2–4 household numbers; adjusted OR = 2.247, 95% CI: 1.105–4.566, *p =* 0.025 for > 4 household numbers), suggesting that household number was associated with acne presentation independent of familial history.

In addition, several lifestyle factors and dietary habits were associated with acne presentation risk. Higher acne risk was found in alcohol drinkers relative to non-drinkers (Table 2). In contrast, lower acne risk was noted in those who consumed butter or drank probiotic drinks on most or all days in the week, relative to those who never or only occasionally did that (Table 2). This suggests a possible protective effects against acne for those who consumed butter and probiotic drinks frequently.

Aside from the above, no significant association with acne presentation was observed for other evaluated factors, including gender, co-morbidities (overweight/obesity, atopy, asthma, rhinitis, eczema, atopic diseases and polycystic ovarian syndrome, PCOS), sedentary lifestyle (increased TV/computer usage and lack of physical activity) and dietary habits of other food groups/type [like meat, fruits, vegetables and high glycaemic index (GI) food; data not shown].

### Familial history strongly influences both lower and higher acne severities and scarring grades compared to controls

As positive familial history of acne was strongly associated with increased risk for acne presentation, we sought to determine whether the same phenomenon could be seen for acne severity and scarring grades. When higher acne severity and scarring grades were compared against lower grades (moderate/severe vs. mild; grade 3/4 vs. grade 1/2), no significant association with familial history of acne was found (except for parental history; data not shown). However, when acne severity and scarring grades were compared against acne controls, strong significant association with maternal, paternal and sibling acne history was found (Table 3). This indicates that those who had acne, regardless of severity and scarring grades, also had a familial history of acne. Furthermore, when maternal and paternal familial history were analysed together as parental familial history, it was observed that having only one parent with acne increased the odds of grade 3/4 acne scarring to 14.925 (Table 3), while having both parents with acne increased the odds of acne to 5.848 (Table 3). Similarly, when parental and sibling familial history were analysed together as familial acne, it was observed that having only one parent/sibling with acne increased the odds of lower acne severity and scarring grades (mild and grade 1/2) to 4.695 and 4.310, and decreased the odds of higher acne severity and scarring grades (severe and grade 3/4) to 3.185 and 6.993, respectively. This suggests that maternal and paternal familial history showed stepwise, additive effects - while sibling history showed stepwise, decrescent effect - on the risk of grade 3/4 acne scarring relative to acne controls.

**Table 3:** Association of familial history with lower and higher grades of acne severity grades and scarring

Variable	Acne severity		Acne scarring	
	Mild vs. controls	Moderate/severe vs. controls	Grade 1/2 vs. controls	Grade 3/4 vs. controls
	OR (95% CI)	OR (95% CI)	OR (95% CI)	OR (95% CI)
**Maternal Acne**				
Yes	2.953 (2.261–3.856)	2.792 (1.925–4.049)	3.068 (2.347–4.012)	3.941 (2.034–7.636)
No	1.00	1.00	1.00	1.00
**Paternal Acne**				
Yes	3.703 (2.788–4.919)	2.611 (1.760–3.875)	3.642 (2.732–4.854)	4.143 (2.135–8.042)
No	1.00	1.00	1.00	1.00
**Parental Acne**				
None	1.00	1.00	1.00	1.00
Only 1 parent	3.509 (2.375–5.208)	3.937 (2.326–6.667)	4.032 (2.717–5.952)	14.925 (6.135–35.714)
Both	3.802 (2.778–5.236)	3.106 (1.984–4.854)	3.774 (2.740–5.208)	5.848 (2.481–13.889)
**Sibling Acne**				
Yes	3.507 (2.722–4.520)	2.521 (1.746–3.639)	3.372 (2.607–4.360)	6.021 (2.748–13.193)
No	1.00	1.00	1.00	1.00
**Familial Acne**				
None	1.00	1.00	1.00	1.00
At least 1 parent/sibling	4.695 (3.546–6.211)	3.185 (2.128–4.762)	4.310 (3.257–5.714)	6.993 (2.890–16.949)

All results were from multivariate analysis on familial history, adjusted fro age and gender

All OR have significance of *p* < 0.001

### Household smoking is a determinant of acne severity, while dietary habits of breakfast food components affect acne scarring extent

Of the lifestyle factors evaluated, smoking and alcohol drinking habits affected acne severity risk. Interestingly, a few of the smoking variables studied were associated with reduced risk for more severe acne. Higher risk for moderate-severe acne was observed in individuals without paternal, parental or household smokers compared to the individuals with those (Table 4), suggesting that having family members who were smokers was associated with a reduced risk of moderate/severe acne. Similarly, having a maternal smoker was associated with a reduced risk of grade 3/4 acne scarring (Table 4). In contrast, other smoking variables studied – personal smoking status and passive smoking – showed no significant association with acne severity and scarring. Alcohol drinking habit was significantly associated with increased risk for higher grades of acne scarring, but this association was abolished after controlling for age and gender (Table 4).

**Table 4:** Association of selected socio-demographic, lifestyle factors, familial history, co-morbidities and dietary habits with acne severity grades and acne scarring grades

Variable	Acne severity				Acne scarring			
	Unadjusted		Adjusted^**a**^		Unadjusted		Adjusted^**a**^	
	**OR (95% CI)**	***p***	**OR (95% CI)**	***p***	**OR (95% CI)**	***p***	**OR (95% CI)**	***p***
***A. Socio-demographic - gender***								
Male	1.2 (0.882–1.634)	0.246	1.225 (0.896–1.675)	0.203	3.244 (1.864–5.644)	< 0.001***	3.238 (1.859–5.639)	< 0.001***
Female	1.00	–	1.00	–	1.00	–	1.00	–
***B. Lifestyle factors***								
**Paternal Smoker**								
Yes	1.00	–	1.00	–	1.00	–	1.00	–
No	1.353 (0.956–1.912)	0.088	1.420 (1.001–2.016)	0.049*	0.901 (0.499–1.626)	0.728	0.919 (0.505–1.675)	0.784
**Maternal Smoker**								
Yes	1.00	–	1.00	–	1.00	–	1.00	–
No	0.561 (0.163–1.933)	0.36	0.582 (0.167–2.03)	0.396	0.305 (0.083–1.126)	0.075	0.210 (0.054–0.813)	0.024*
**Parental Smoking**								
None	1.375 (0.972–1.944)	0.072	1.450 (1.022–2.058)	0.038*	0.956 (0.509–1.795)	0.888	0.912 (0.480–1.733)	0.780
At least 1	1.00	–	1.00	–	1.00	–	1.00	–
**Household smokers**								
None	1.449 (1.030–2.039)	0.033*	1.529 (1.083–2.159)	0.016*	0.980 (0.542–1.775)	0.948	0.985 (0.539–1.801)	0.962
At least 1	1.00	–	1.00	–	1.00	–	1.00	–
**Alcohol drinking**								
Non-drinker	1.00	–	1.00	–	1.00	–	1.00	–
Drinker	1.183 (0.877–1.596)	0.272	1.218 (0.895–1.657)	0.209	1.821 (1.032–3.215)	0.039*	1.555 (0.869–2.786)	0.137
***C. Familial history***								
**Parental Acne**								
None	1.00	–	1.00	–	1.00	–	1.00	–
Only 1 parent	0.824 (0.506–1.341)	0.436	0.872 (0.531–1.432)	0.587	3.049 (1.346–6.944)	0.008**	3.817 (1.634–8.929)	0.002**
Both	1.101 (0.718–1.689)	0.659	1.195 (0.773–1.847)	0.423	1.572 (0.687–3.597)	0.284	1.515 (0.653–3.521)	0.333
***D. Co-morbidities***								
**BMI**								
18.5–23	1.00	–	1.00	–	1.00	–	1.00	–
< 18.5	1.099 (0.721–1.675)	0.659	1.146 (0.748–1.755)	0.531	2.404 (1.185–4.878)	0.015*	2.445 (1.186–5.051)	0.015*
> 23	1.061 (0.754–1.494)	0.735	0.957 (0.674–1.36)	0.808	1.381 (0.731–2.611)	0.320	1.241 (0.648–2.375)	0.515
**Asthma**								
Yes	0.631 (0.313–1.27)	0.197	0.544 (0.263–1.128)	0.102	4.359 (1.107–17.172)	0.035*	2.945 (0.689–12.588)	0.145
No	1.00	–	1.00	–	1.00	–	1.00	–
***E. Dietary habits***								
**Nuts**								
Never/ occasionally	1.00	–	1.00	–	1.00	–	1.00	–
Once or twice/week	0.562 (0.384–0.824)	0.003**	0.585 (0.396–0.864)	0.007**	0.808 (0.404–1.618)	0.547	0.659 (0.322–1.346)	0.252
Most or all days	0.524 (0.268–1.022)	0.058	0.547 (0.278–1.076)	0.081	0.646 (0.186–2.247)	0.492	0.692 (0.195–2.451)	0.569
**Burgers/fast food**								
Never/ occasionally	1.00	–	1.00	–	1.00	–	1.00	–
Once or twice/week	0.637 (0.464–0.875)	0.005**	0.603 (0.436–0.834)	0.002**	0.746 (0.425–1.309)	0.307	0.660 (0.371–1.174)	0.157
Most or all days	0.876 (0.500–1.534)	0.642	0.836 (0.473–1.477)	0.537	0.726 (0.243–2.174)	0.567	0.745 (0.245–2.262)	0.604
**Cereal**								
Never/ occasionally	1.00	–	1.00	–	1.00	–	1.00	–
Once or twice/week	1.022 (0.587–1.781)	0.938	0.943 (0.537–1.655)	0.838	0.536 (0.231–1.244)	0.146	0.437 (0.183–1.046)	0.063
Most or all days	1.005 (0.569–1.776)	0.986	0.956 (0.537–1.701)	0.877	0.303 (0.117–0.788)	0.014*	0.278 (0.105–0.739)	0.010*
**Butter**								
Never/ occasionally	1.00	–	1.00	–	1.00	–	1.00	–
More than once/week	0.858 (0.599–1.229)	0.403	0.835 (0.578–1.205)	0.335	0.504 (0.260–0.975)	0.042*	0.451 (0.230–0.887)	0.021*
**Milk**								
Never/ occasionally	1.00	–	1.00	–	1.00	–	1.00	–
Once or twice/week	1.33 (0.946–1.869)	0.101	1.294 (0.916–1.828)	0.144	0.457 (0.255–0.816)	0.008**	0.441 (0.244–0.797)	0.007**
Most or all days	1.095 (0.724–1.655)	0.667	1.111 (0.731–1.69)	0.622	0.232 (0.088–0.609)	0.003**	0.259 (0.097–0.689)	0.007**

Results are from univariate and multivariate analysis on the variables associated with moderate/severe acne with reference to mild acne, or acne cases with Grade 3/4 scarring with reference to acne cases with Grade 1/2 scarring

Values are odds ratio (95% CI); *p < 0.05 ** *p* < 0.01 *** *p* < 0.001

^a^Adjusted for age and gender

In addition, dietary habits of certain food groups/types were also associated with protective effects for moderate/severe or grade 3/4 acne. Consumption of nuts and burgers/fast food once/twice per week was associated with lower risk for higher acne severity, while more frequent consumption (once/twice per week and above) of cereals, butter and milk (which seems to be food components commonly consumed during breakfast) was associated with lower risk for higher acne scarring (Table 4). Lower odds ratios of down to 0.26 were seen for consumption of cereals and milk for most or every day of the week, suggesting that frequent consumption of cereals with milk for breakfast particularly reduces the risk for higher acne scarring. Further comparison between acne grade 3/4 vs. acne controls similarly replicated this significant association; with adjusted OR of 0.240 (95% CI: 0.091–0.637; *p* = 0.004) and 0.129 (95% CI: 0.017–0.983; *p* = 0.048) for consumption of milk and butter for most or every day of the week, respectively.

Apart from that, being male, underweight and having current asthma were associated with higher grades of acne scarring (Table 4). Further comparison between acne grade 3/4 vs. acne controls also replicated the significant increase in higher acne scarring risk among males (adjusted OR = 3.411; 95% CI: 1.958–5.944; *p* < 0.001) and underweights (adjusted OR = 2.160; 95% CI: 1.053–4.425; *p* = 0.036).

## Discussion

In the Malaysian Chinese studied, the prevalence of acne was 60.7%, lower than the prevalence among similar studies in Malaysia among Chinese adolescents (72.5% [12];) and medical students (68.1% [13];). The observed prevalence of moderate/severe acne (21.6%) and acne scarring (69.9%) was higher than the prevalence in previous Malaysian studies (9.8% [12]; and 9.3% [13]; for moderate/severe acne; 59% for acne scarring, [13]). Finally, while we did not find the male gender as a predisposing risk factor for acne presentation and severity unlike previous Malaysian studies [12, 13], we found that males had an increased risk for higher grades of acne scarring.

In this study, we found that those who had acne, regardless of severity and scarring grades, had strong positive familial history (either in parents and/or sibling). This proves that acne is a highly heritable trait. Recent genome-wide association studies (GWAS) and candidate gene association studies have identified that genetics, particularly gene variations, play a strong role in acne pathogenesis by modulating biological pathways like androgen metabolism, inflammation, stem cell fate and tissue remodelling (reviewed in [17]). Familial history did not show an additive effect for acne presentation; but for higher grades of acne scarring, maternal and paternal familial history showed stepwise, additive effects, while sibling history showed the opposite effect. However, in another Malaysian study, while maternal and paternal acne history significantly contributed to odds for developing acne (1.752 and 1.852, respectively), parental history showed an additive effect as the odds was increased to 3.056 [13]. Our recent meta-analysis found a pooled odds ratio of 2.91 (95% CI 2.58–3.28; familial history in parents with reference to no familial history in parents [9]); for acne presentation. However, the association between familial history and acne severity and scarring is unclear; some studies reported a higher prevalence of severe acne grade in those with a positive familial history of acne [18], while others did not [19–21].

Dietary habits of certain food types/group seemed to decrease the risk for acne presentation, severity and scarring. Frequent consumption (most or all days) of foods that are commonly consumed during breakfast (butter, probiotic drinks, cereals and milk) decreased the risk for acne presentation and higher acne scarring, while periodic consumption (once/twice per week) of nuts and burgers/fast food decreased the risk for higher acne severity. This seems to indicate that having breakfast that is composed of those food components reduced acne presentation and higher scarring grade risks among Malaysian Chinese. In contrast, Ismail et al. [10] found that the consumption of milk ≥ once a week increased the risk of acne occurrence by 4 times (OR = 3.99, 95% CI =1.39–11.43) in a predominantly Malay Malaysian study, while Suppiah et al. [11] found that milk consumption was significantly higher (OR = 2.19, 95% CI = 1.04–4.65) among acne cases in a predominantly Chinese Malaysian study. The potential link between diet and acne presentation has been widely debated, with some studies finding no significant diet-acne link and others reporting that the intake of certain foods is associated with acne presentation (reviewed in [9]). For example, several studies found that frequent consumption of certain dairy products [22–24], carbohydrates, high GI and glycaemic load (GL) diet [25–27] is a potential risk factor for acne, which could be attributed partly to the insulin-like growth factor 1 (IGF1)/FoxO1/mTORC1 signalling pathway. Meanwhile, high adherence to a Mediterranean diet - characterised by an omega-3 rich, low-GI diet – was associated with a protective effect against acne, possibly by anti-inflammatory and anti-IGF1 mechanisms [28]. Probiotic use, either through topical or oral administration, has been shown to inhibit acne pathogenesis by directly inhibiting *C. acnes* through the production of antibacterial proteins or through anti-inflammatory immunomodulatory effects (reviewed in [29]). However, neither earlier Malaysian studies found significant association between yoghurt intake and acne risk [10, 11]. Taken together, since Jung et al. [30] showed that controls had higher regular breakfast intake compared to acne patients among Koreans, this indicates that regular breakfast intake - probably composing of butter, probiotic drink, cereals and milk as shown in this study - helps in reducing risk for acne presentation and higher acne scarring.

A few smoking variables – having a father who smokes, having at least one smoking parent and living in a household with at least one smoker – were associated with reduced risk of more severe acne in this Malaysian Chinese population. However, other smoking variables – personal smoking status and passive smoking – were not associated with acne severity, similar with an earlier Malaysian study [11]. We recently reviewed that the link between smoking and acne severity is controversial, with inconsistent results between studies [9]. Rombouts et al. [31] observed that smoking was linked to decreased acne risk in girls, while smoking was found to be a risk factor for acne [20] and severe acne occurred more frequently among smokers [32]. Studies also considered smoking duration and number of cigarettes smoked - one study observed a significant dose-dependent relationship between acne severity and the number of cigarettes smoked per day [33], another did not [31]. The complex way in which smoking influences the pathogenesis of acne including inflammation, wound healing and immune responses [31], may contribute, in part, to these inconclusive evidences.

In this study, we found that alcohol drinking was significantly associated with increased risk for acne presentation and higher grades of acne scarring, but the latter association was abolished after controlling for age and gender. In our recent review, alcohol intake is consistently not significantly associated with acne risk [9], as shown by majority negative previous findings [18, 33–36].

BMI, sedentary lifestyle (assessed via computer/television usage), atopy, asthma, allergic rhinitis, eczema, atopic diseases and PCOS were not significantly associated with acne presentation in this Malaysian Chinese population. Nevertheless, being underweight was associated with increased risk of having higher grades of acne scarring, contrasting the findings of previous studies that reported higher acne prevalence in overweight individuals [18–20, 37–39]. However, an earlier Malaysian study found no significant association between BMI and acne presentation [10]. We also reported that having current asthma is a predisposing risk factor for higher grades of acne scarring, similar with the finding by Silverberg and Silverberg [40] which reported a significant association of severe acne with asthma.

Finally, age and household number were significantly associated with acne risk among Malaysian Chinese. Previous reports have observed highest acne prevalence among older teenagers and young adults and lower acne prevalence among older adults [41], which is in line with the results obtained in this study that demonstrate a higher risk of acne in those aged 20–24 relative to those > 24. None of the studies reviewed by us recently [9] investigated the association between household number and acne risk; thus, further studies are needed to understand the true relationship between the two.

We acknowledge a few limitations in our study. First, as the study subjects were Chinese university students and staff, the findings of this study could not be extrapolated to the multi-ethnic Malaysian population. Next, due to the retrospective cross-sectional study design used, it could only determine the differences within a population between those who have developed acne and those who have not. It was also only able to determine the association, but not the cause and effect of the modifiable risk factors on acne. Also, following the removal/addition of some list of foods in the questionnaire, we have not performed a reliability and validity calibration assessment. Nevertheless, removal/addition of foods is actually recommended by the original questionnaire designers in order to make it more relevant for a particular country [14]. Apart from the three-level food frequency questionnaire used in this study, more frequency levels, more commonly-consumed food types, or even a repeated three-day food dairy (two weekdays and one weekend) could be adopted. Other psychosocial factors contributing to - or impacted from acne - like quality of life [12, 13], stress, depression, anxiety, and sleep quality, could be taken into account in future studies. Despite these limitations - with sample size of almost 2000 subjects – we have evaluated a wide range of epidemiological risk factors and encompassing not just acne presentation, but also severity and scarring. The findings of from our study have provided further support to the existing evidence for the role of familial history and dietary habits in acne occurrence.

## Conclusions

In conclusion, positive familial history is a strong predisposing factor in influencing acne presentation, severity and scarring among Malaysian Chinese. Frequent consumption of foods that are commonly consumed during breakfast (butter, probiotic drinks, cereals and milk) is protective against presenting with acne and having higher acne scarring grades, while alcohol drinking habit is a predisposing risk factor for acne among Malaysian Chinese. The predisposing risk factors revealed in this study can help researchers and clinicians to understand the epidemiology and pathophysiology of acne, and hence develop effective interventions especially targeting modifiable factors like dietary habits and lifestyle factors.

## Supplementary Information

**Additional file 1.**

## Data Availability

All data used and included in this study are available from the corresponding author (F.T.C.) on reasonable request.

## Abbreviations

BMI: Body mass index
CI: Confidence interval
GI: Glycaemic index
GL: Glycaemic load
OR: Odds ratio
SD: Standard deviation
