# Supplementary material for: Modifiable and non-modifiable epidemiological risk factors for acne, acne severity and acne scarring among Malaysian Chinese: a cross-sectional study
Source: BMC Public Health. 2021 Mar 27;21:601. doi: 10.1186/s12889-021-10681-4 (PMC8005239; doi:10.1186/s12889-021-10681-4)
Supplement: Supplementary file 1 — Additional file 1. [file 12889_2021_10681_MOESM1_ESM.docx]

**Title: Modifiable and non-modifiable epidemiological risk factors for acne, acne severity and acne scarring among Malaysian Chinese: a cross-sectional study**

Yee-How Say^a^, Anna Hwee Sing Heng^a^, Kavita Reginald^b^, Yi Ru Wong^b^, Keng Foo Teh^b^, Smyrna Moti Rawanan Shah^b^, Yang Yie Sio^a^, Yu Ting Ng^a^, Sri Anusha Matta^a^, Sze Lei Pang^a^, Fook Tim Chew^a*^

^a^Department of Biological Sciences, Faculty of Science, National University of Singapore, Singapore

^b^Department of Biological Sciences, School of Science and Technology, Sunway University, Malaysia

**Supplementary Information**

**Additional file 1: Questionnaires used in this study**

As this study was part of an ongoing epidemiological study on allergic diseases such as asthma, atopic dermatitis and allergic rhinitis, we adapted the validated questionnaire from the Global Asthma Network (Ellwood P, Asher M, Ellwood E, Global Asthma Network Steering Group. Manual for Global Surveillance: Prevalence, Severity and Risk Factors. Auckland, New Zealand: Global Asthma Network Data Centre, 2015) and our group’s previous published study (Zuraimi MS, Tham KW, Chew FT, Ooi PL, David K. Home exposures to environmental tobacco smoke and allergic symptoms among young children in Singapore. Int Arch Allergy Immunol. 2008;146(1):57-65). Particularly, some of the list of foods in the Global Asthma Network questionnaire were deleted and some relevant examples relevant for Malaysia were added.

**Sunway University data collection questionnaire**

**Core questionnaire for acne**

1. Have you ever had acne? Yes/No

2. At what age did you first have acne? (age)_________

3. Have you had acne in the last 12 months? Yes/No

4. Have you ever visited a doctor for your acne condition? Yes/No. If "no", skip to question 9

5. Were you given any of the following treatment for acne? (tick all that apply) None, Pills, Creams, Others:_________

6. If you were given 'Pills', please indicate the name/ type (tick all that apply) Retinoids (Vitamin A), Antibiotics, Chinese Medicine, Others:_________

7. If you were given 'Creams', please indicate the name/ type (tick all that apply) Retinoids (Vitamin A), Antibiotics, Chinese Medicine, Benzoyl peroxide/ resorcinol, Others:_________

8. Did your acne get better after treatment? Yes/No

9. Have you ever had black-heads? Yes/No

10. Have you ever had white-heads? Yes/No

11. Which of the following body sites had/ have red swollen boils/ yellow heads or acne?

(tick all that apply) Face and neck, Chest and back, Arms and legs

12. Do you have scars (keloids) left by acne/boils? Yes/No

13. Has your mother ever been diagnosed with Acne (Pimples)? Yes, No, Don’t know

14. Has your father ever been diagnosed with Acne (Pimples)? Yes, No, Don’t know

15. How many of your siblings have been diagnosed with the Acne (Pimples)? 0, 1, 2, 3 or

more, Don’t Know

**Questionnaire on demographic, socioeconomic and household factors**

1. Gender: Male, Female

2. Age: _______

3. Date of birth: _____________ (dd/mm/yy)

4. Race: Malay, Chinese, Indian, Other: ________

5. Country of birth: Malaysia, Other: ________

6. Number of years in Malaysia: _________

7. Type of Housing: Flats, Condominium/Private Apartments, Landed Property

8. Total monthly family income: < RM3000, RM3000-5999, RM6000-12999, >RM13000

9. Number of people in household (including yourself): ______

10. What is the highest level of education completed by your mother? Primary, Secondary, Tertiary (Diploma & above)

11. What is the highest level of education completed by your father? Primary, Secondary, Tertiary (Diploma & above)

12. Please state the number of siblings that you have (excluding yourself): _________

**Questionnaire on personal health history**

1. Weight (in kg): ____________

2. Height (in cm): ____________

3. Do you have any drug allergies? Yes/No

4. At what age did you start menstruating? _________

5. For female participants: Menstrual cycle: Regular, Irregular

6. For female participants: History of polycystic ovarian syndrome? Yes, No, Don’t know

7. Do you consume any Oral contraceptives? Yes/No

A modified version of the International Study of Asthma and Allergies in Childhood (ISAAC) written questionnaire was used to determine asthma, eczema and rhinitis status. In addition, a skin prick test was conducted to determine atopy status.

**Questionnaire on dietary, smoking and lifestyle factors**

1. In the past 12 months, how often, on average, did you eat or drink the following: Meat (e.g. Beef, lamb, chicken, pork); Seafood (including fish); Fruits; Vegetables (green and root); Pulses (peas, beans, lentils); Cereals (including bread); Pasta; Rice; Butter; Margarine; Nuts; Potatoes; Milk; Eggs; Burgers/ fast food; Yakult/Vitagen/similar yogurt drinks? Never or only occasionally, Once or Twice per week, Most or All days

2. How many times a week do you engage in vigorous physical activity long enough to make you breathe hard? Once or twice per week, Most or all days, Never or only occasionally

3. How many hours of do you spend in front of the television or computer every day? Less than 1 hour, 1 to 3 hours, More than 3 hours to 5 hours, More than 5 hours

4. How often do you consume alcohol? Frequent, Occasional, Non-drinker

5.

a. What is your smoking status? Smoker, Ex-smoker, Non-smoker

b. If you currently are a smoker,

How many years have you been smoking? ______________

How many cigarettes do you smoke every day? ______________

c. If you have already stopped smoking,

How many years has it been since you stopped smoking? ______________

For how many years did you use to smoke? ______________

How many cigarettes did you use to smoke every day? ______________

6. Please state the number of people living in the household who smoke cigarettes,

including parents: _________

7. Does your father (or male guardian) smoke cigarettes? Yes/No

If YES, please state the amount of cigarettes which your father (or male guardian)

smoke each day: Number of Cigarettes______ /day

8. Does your mother (or female guardian) smoke cigarettes? Yes/No

If YES, please state the amount of cigarettes that your mother (or female guardian)

smoke each day: Number of Cigarettes______ /day

9. Does anyone living with you smoke cigarettes in your presence? Yes/No

10. Have you ever had animals (e.g. cats, dogs, mice, hamsters, etc) in your house? Yes/No

11. At what AGE did you have the following animals (Cat; Dog; Rodent (hamster, mouse,

etc.); Birds (chicken, parrot, pigeon, etc.); Other: ______) in your house? (tick all that

apply) <2, 2-6, 7-12, 13-18, >18

**Universiti Tunku Abdul Rahman (UTAR) data collection questionnaire**

The questionnaires administered to participants from UTAR and Sunway University were

very similar. The questions in both surveys were identical, except for the differences

highlighted below.

**Core questionnaire for acne**

1. Have you ever had acne? Yes/No If "yes", age first had acne _______

2. If you were given 'Creams', please indicate the name/ type (tick all that apply)

Retinoids (Vitamin A), Antibiotics, Chinese Medicine, Others:_________

3. Has your brother/sister ever been diagnosed with Acne (Pimples)? Yes, No, Don’t

know

**Anthropometric measurements**

1. Systolic Blood Pressure (mmHg): ______

2. Diastolic Blood Pressure (mmHg): ______

3. Pulse rate (bpm): ______

4. Waist circumference (cm): ______

5. Hip circumference (cm): ______

6. BMI (kg/m2): ______

7. TBF (%): ______

8. SF (%): ______

9. VFL (%): ______

10. SM (%): ______

56

11. RM (kcal) : ______

**Questionnaire on demographic, socioeconomic and household factors**

1. Race: Malay, Chinese, Indian

2. Which state did you grow up in until age 15? (e.g. Perak; if not in Malaysia, please write country) ________

3. Number of years in Malaysia: _________

4. Type of Housing: Flats, Condominium/Private Apartments, Landed Property

5. What is your family’s household income category? (Median household income for 2016 are shown in brackets): T20 (RM13,148), M40 (RM6,275), B40 (RM3,000)

6. How many people were in your household when you were growing up? ______

7. Question 12 from the Sunway survey were not asked in the UTAR survey

**Questionnaire on personal health history, dietary, smoking and lifestyle factors**

1. At what age did you start menstruating? <10 years old, 10-14 years old, >14 years old

2. In the past 12 months, how often, on average, did you eat or drink the following: Sweets; Chocolates? Never or only occasionally, Once or Twice per week, Most or All days

3. How many times a week do you engage in vigorous physical activity long enough to make you breathe hard? Never/occasionally, 1-2 times/week, ≥3 times/week

4. How many people living in your house smoke cigarettes? _________

5. Does anyone living with you smoke cigarettes in your presence? Yes/No

6. In the past 12 months, have you ever had a cat and/or dog in your house? Dog, Cat, Dog & Cat, No

7. Questions 5b, 5c, 7 (If YES) and 8 (If YES) from the Sunway survey were not asked in the UTAR survey
